# Predictors of tuberculosis preventive therapy initiation among people living with HIV in the Volta region, Ghana

**DOI:** 10.1186/s12889-026-28511-w

**Published:** 2026-07-23

**Authors:** Felix Mbiba, Raham Agbenorku Yaw Tawiah, Joyce Berkumwin Der

**Affiliations:** Department of Epidemiology and Biostatistics, Fred N. Binka School of Public Health, University of Health and Allied Sciences, Hohoe, Ghana

**Keywords:** Tuberculosis preventive therapy, People living with HIV, HIV care, Tuberculosis prevention, Treatment uptake, Ghana

## Abstract

**Background:**

Tuberculosis (TB) remains a leading cause of morbidity and mortality among people living with HIV (PLHIV) despite the availability of effective preventive interventions. Tuberculosis Preventive Therapy (TPT), strongly recommended by the World Health Organization, substantially reduces the risk of active TB among PLHIV, however, uptake of TPT remains suboptimal in many settings. This study, therefore, assessed the predictors of TPT initiation, beginning the course of anti-TB medications among PLHIV.

**Methods:**

This was a health facility-based analytical cross-sectional study among 806 PLHIV aged *≥* 18 years attending antiretroviral therapy (ART) clinics in the Volta Region of Ghana. A systematic sampling technique was employed to recruit respondents, and data were collected using structured questionnaires mounted in the Kobo Toolbox. Data was exported to Stata version 17.0 for analysis. Descriptive and logistic regression analyses were performed, with statistical significance set at a *p* < 0.05 at 95% confidence interval.

**Results:**

Our study found that out of the 806 PLHIV recruited, with a mean age of 44.7 years (SD ± 13), the majority, 481 (59.70%), were found to have poor knowledge of TPT. We found that 620/806 (88.6%) PLHIV were eligible for TPT, and among those eligible, only 236 (38.1%) were initiated on TPT. Respondents who had good knowledge of TPT were about 7 times more likely to be initiated compared with those who had poor knowledge (aOR = 6.79, 95% CI 4.69–9.83, *p* < 0.001). Those who had been diagnosed with HIV for one year or more were 4 times as likely to be initiated as those diagnosed for less than a year (aOR = 4.18, 95% CI 1.74–10.04, *p* = 0.001). Respondents in HIV clinical stage 2 were 66% less likely to be initiated than those in stage 1 (aOR = 0.34, 95% CI 0.20–0.58, *p* < 0.001). With respect to marital status, married respondents were 40% less likely to be initiated on TPT compared with single respondents (aOR = 0.60, 95% CI 0.37–0.97, *p* = 0.038). Barriers perceived by PLHIV to TPT initiation were distance to facility, poor health education, and limited drug availability.

**Conclusion:**

TPT initiation among PLHIV in the Volta Region was suboptimal, and key predictors of initiation were TPT knowledge, duration since HIV diagnosis, HIV clinical stage, and marital status. Strengthening patient education, ensuring consistent drug and logistics availability, and addressing implementation barriers are critical to improving uptake and advancing progress toward Sustainable Development Goal 3.3 targets.

**Supplementary Information:**

The online version contains supplementary material available at https://doi.org/10.1186/s12889-026-28511-w.

## Background

Tuberculosis (TB) remains the leading cause of death from a single infectious agent, with an estimated global total of 10.7 million people falling ill with TB in 2024, equivalent to 131 incident cases per 100 000 population, with an estimated 1.08 million deaths among HIV-negative people and an estimated 150,000 deaths among PLHIV, with a combined total of 1.23 million [1]. TB is the most common opportunistic infection with a high mortality rate among HIV-infected individuals. The risk of TB in HIV-infected persons continues to increase as HIV disease progresses and immunity decreases [2]. Compared to those who are HIV negative, who have a lifetime risk of roughly 10–20%, people living with HIV (PLHIV) have a yearly risk of 5–10% of developing active TB disease [3]. In an effort to prevent and lower the incidence of TB among people living with HIV (PLHIV), the World Health Organization (WHO) recommends TB preventive therapy (TPT). TPT is the Treatment offered to individuals who are considered at risk of TB disease to reduce that risk [4]. It is one of the key interventions recommended by WHO to achieve the End TB Strategy targets of reducing TB deaths and incidence rates by 75% and 50%, respectively, by 2025 compared to 2015 rates [5]. TPT is a key component of HIV care because it has a synergistic effect with Antiretroviral Therapy (ART) and also independently lowers the risk of TB disease and has been shown to reduce the incidence of TB disease among PLHIV by 33%–62% [6, 7].

Despite the well-documented benefits of TPT, its implementation remains suboptimal in many settings [8]. In 2023, the global coverage of TPT among PLHIV was 56% below the 90% target [1]. In Ghana, the national TB control programme rolled out the implementation of TPT in 2021, however, as of 2023, the uptake of TPT among PLHIV is unknown [9]. Several factors hinder the implementation and uptake of TPT, such as inadequate monitoring, skepticism about TPT effectiveness, and resource shortages [10, 11]. Identifying and addressing challenges associated with the implementation and uptake of TPT is crucial to improving the health outcomes of PLHIV and reducing the burden of TB in this population. This study, therefore, determined the predictors of TPT initiation among PLHIV in the Volta region of Ghana.

## Materials and methods

We adopted Strengthening the Reporting of Observational studies in Epidemiology (STROBE) guidelines [12]. The study methodology consists of the study design, study site, study population, sampling method, data collection, study variables, quality control, data analysis, and ethical considerations.

### Study design

The study adopted a health facility-based analytical cross-sectional design to determine the knowledge, patient-related, and healthcare system-related factors associated with the initiation of TPT among PLHIV in the Volta region of Ghana. The design was cost-effective, relatively easy to implement, and allowed for the simultaneous analysis of multiple variables. The design helped to collect data from respondents at one point in time. Data was collected from PLHIV at their various ART clinics.

### Study site description

**Fig. 1:**
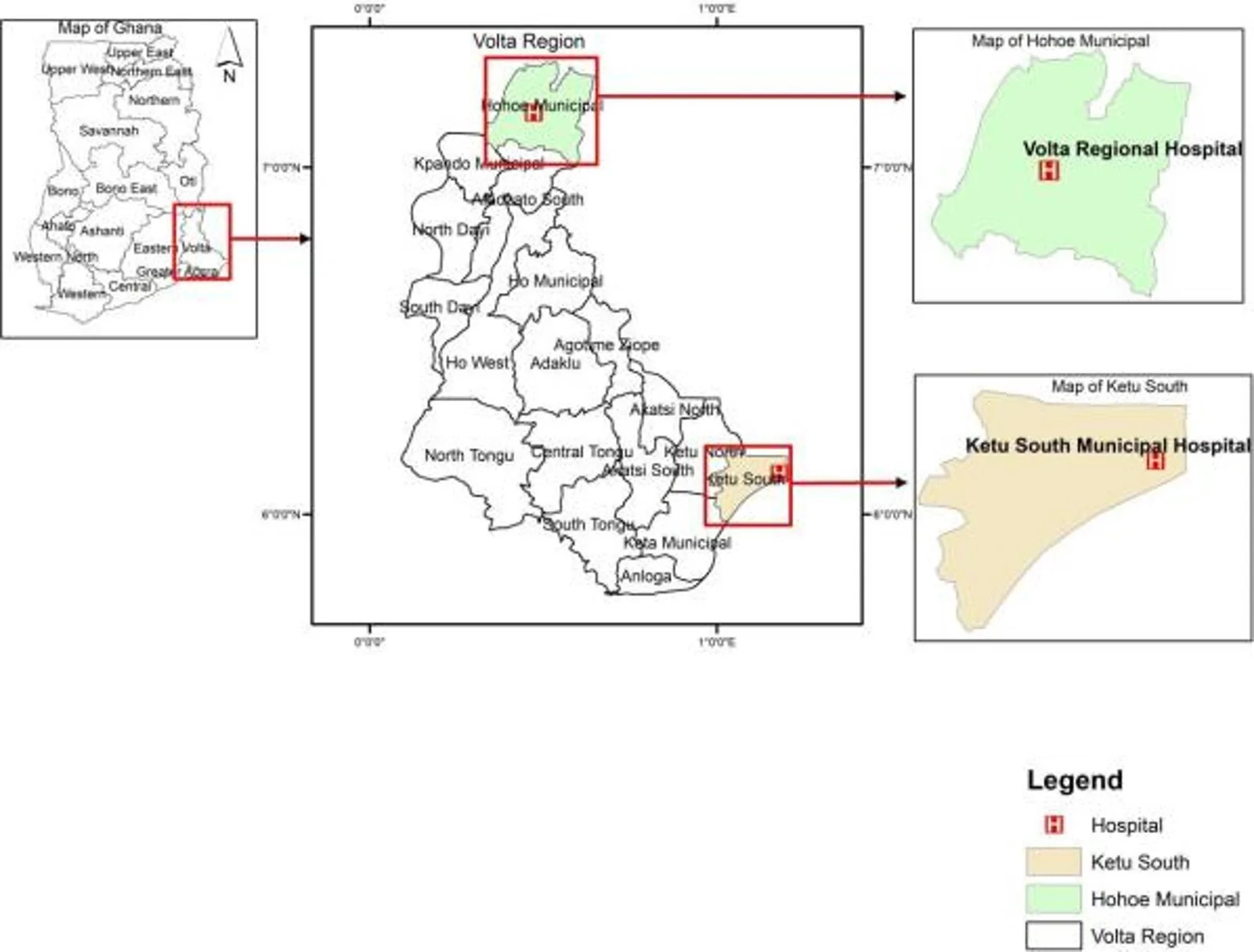
Study area Map

The study was conducted in the Hohoe and Ketu South Municipalities, located in the northern and southern parts of the Volta region, respectively. The health facilities selected for the study were the Volta Regional Hospital (VRH) in Hohoe and the Ketu South Municipal Hospital (KSMH) in Ketu South. These facilities were purposively chosen because they host accredited ART clinics and serve as major referral centers for HIV care within their respective municipalities and adjoining districts. Their roles in the delivery of comprehensive HIV services made them appropriate sites for the present study. The VRH, located in Hohoe, functions as a secondary-level referral facility within the regional health system. It provides a broad range of clinical services, including outpatient and inpatient care, maternal and child health services, surgical services, laboratory diagnostics, and specialized HIV care. The hospital operates an established ART clinic that delivers comprehensive HIV-related services such as testing and counseling, initiation and monitoring of antiretroviral therapy, adherence counseling, and management of opportunistic infections. The ART clinic at VRH runs dedicated sessions once weekly, during which clients receive routine clinical reviews, medication refills, and laboratory follow-up as indicated. The hospital serves a diverse patient population drawn from Hohoe Municipality and neighboring districts, including cross-border clients from Togo. Similarly, the KSMH is a secondary-level public health facility located in the municipal capital. The hospital provides general medical services alongside specialized programs, including HIV prevention, treatment, and care. The ART clinic at KSMH is structured to enhance access to care for clients within the municipality and surrounding communities. Unlike VRH, the ART clinic at KSMH operates twice weekly, thereby accommodating a larger patient flow and improving continuity of care. Services offered at the clinic include HIV testing and counseling, clinical staging and monitoring, antiretroviral drug dispensation, adherence support, and routine laboratory investigations. Both facilities operate under the Ghana Health Service (GHS) framework and adhere to national guidelines for HIV management [13]. Their established ART clinics, structured clinic days, and consistent patient attendance provided a stable and organized environment for data collection. The selection of these two hospitals, therefore, ensured representation of both northern and southern parts of the Volta Region while maintaining methodological consistency in terms of service delivery structure and HIV care provision. (Fig. 1)

### Study population

Our study was conducted among PLHIV in the Volta region. Specifically, respondents were eligible for inclusion in the study if they were aged 18 years or older, had a confirmed HIV-positive diagnosis, were enrolled in HIV care, and were receiving treatment at the ART clinic of the VRH or KSMH. Clients who were severely ill to participate in an interview and those were excluded.

### Sample size determination

The minimum sample size for this study was determined using the single proportion formula by Cochran (1977),

$$\:\mathbf{n}=\frac{{\boldsymbol{Z}}^{2}\boldsymbol{P}(1-\boldsymbol{P})}{{\boldsymbol{e}}^{2}}$$

First, calculate each component:

$$\:\left(1.96)^{2}=3.8416\right.$$

$$\begin{aligned}\:0.50(1-0.50)=\\0.50\times\:0.50=0.25\end{aligned}$$

$$\:\left(0.05)^{2}=0.0025\right.$$

Substituting:

$$\:{n}_{0}=\frac{3.8416\times\:0.25}{0.0025}$$$$\:{n}_{0}=\frac{0.9604}{0.0025}$$$$\:{n}_{0}=384.16$$To account for potential non-response or incomplete data, a 5% non-response rate was considered.

$$\left(\text384\times0.05\right)+\text384=\text403.2$$

Assuming a 95% confidence level (Z = 1.96), a 5% margin of error (e = 0.05), and a conservative population proportion of 50% (*P* = 0.50) due to the absence of prior estimates, the calculated minimum sample size was 384 respondents. After adjusting for a 5% non-response rate, the final minimum sample size per facility was approximately 403 respondents. Consequently, a total of 806 respondents were recruited, comprising 403 respondents from the VRH and 403 respondents from the KSMH.

### Sampling technique

A systematic sampling technique was employed to select study respondents from the ART clinics of VRH and the KSMH. This probability sampling approach was considered appropriate because it ensured an even distribution of respondents across the sampling frame while maintaining operational feasibility within the structured clinic environment. The sampling frame for each facility was obtained by reviewing the official ART clinic registers. These registers contain the unique identification numbers and records of all PLHIV who were actively receiving care at the time of data collection. A review of the registers indicated that 753 clients were actively registered at the ART clinic of the VRH and 1,150 clients were actively registered at the ART clinic of the KSMH. These figures constituted the accessible study population in each facility and served as the basis for calculating the sampling interval. The sampling interval was determined separately for each facility by dividing the number of clients registered in each ART register by the required sample size for that facility. This yielded a sampling interval of approximately 2 for the VRH (753/403 = 2) and 3 for the KSMH (1,150/403 = 3). Approximately every second and third eligible client at the VRH and KSMH, respectively, was selected from the register during the recruitment period. At the beginning of data collection in each facility, a random starting point was determined. This was achieved by selecting a number at random, (2) for VRH and (1) for KSMH. The patient corresponding to the selected number (2 and 1) in the ART register served as the first participant.

Subsequently, every second and third client on the register for VRH and KSMH, respectively, was approached for recruitment. On clinic days, selected clients were identified as they reported for their scheduled visits. Eligibility was confirmed, and the purpose of the study was explained to them. Written informed consent was obtained from clients who agreed to participate. If a selected client declined participation or did not meet the inclusion criteria, the next eligible client on the register was approached to maintain the sampling interval and minimize selection bias. This process continued sequentially until the required sample size of 403 respondents per facility was attained.

### Data collection instrument and procedure

Data collection was conducted over a period of one month, from June to July 2023. A structured questionnaire was developed to gather detailed information on respondents’ socio-demographic characteristics, clinical characteristics, as well as patient-related and health system-related factors influencing the implementation and initiation of TPT. The questionnaire was carefully structured to ensure clarity, logical flow, and relevance to the study objectives. Before the commencement of data collection, research assistants were trained on the study objectives, ethical considerations, interview techniques, and the use of electronic data collection tools. This training ensured uniformity in data collection procedures and minimized interviewer bias. Eligible respondents were identified during their routine ART clinic visits. After eligibility screening, the study was explained to respondents in clear terms, and written informed consent was obtained before their participation. The questionnaire was then administered through face-to-face interviews in a private setting within the health facility to ensure confidentiality and encourage accurate responses. For respondents who were selected but did not attend the clinic during the data collection period, follow-up phone calls were made using contact information available in the clinic register to schedule or book appointments with them for the interview. This approach helped reduce non-response and ensured adequate representation of the study population. The questionnaire was programmed using Kobo Toolbox to enhance data quality through built-in validation checks, skip patterns, and compulsory response fields. Data were collected electronically on mobile devices using the Kobo Toolbox. The use of electronic data capture minimized data entry errors, eliminated the need for manual data transcription, and enabled real-time monitoring of data completeness. The study, therefore, drew on two complementary data sources. Information on socio-demographic characteristics, knowledge of TPT, perceived barriers, and personal experiences with TPT services was obtained directly from respondents through the interviewer-administered questionnaire and was therefore self-reported. In contrast, clinical information, including TPT initiation status, WHO HIV clinical stage, duration since HIV diagnosis, duration on ART, and tuberculosis screening and eligibility status, was abstracted from the respondents’ clinical folders and ART registers. Where clinical details were also captured during the interview, they were cross-checked against the documented records to ensure accuracy and minimize recall bias. The questionnaire used for this study was adapted and contextualized from similar studies [14–17] (Supplementary file). 

## Study variables

### Dependent variable

The dependent variable for this study was TPT initiation status. TPT initiation was defined as documented evidence in the patient’s clinical folder or ART register indicating that the patient had commenced TPT at any point during HIV care. This outcome was measured as a binary variable and coded as “1” for patients who had been initiated on TPT and “0” for those who had not. The classification relied primarily on documented clinical records to ensure accuracy and minimize recall bias, with information obtained from interviews cross-checked against patient folders where necessary.

### Independent variables

The independent variables were grouped into three main domains: socio-demographic characteristics, clinical and HIV-related information, and knowledge. Socio-demographic variables captured respondents’ personal and social attributes. These included age, sex, ethnicity, religion, marital status, educational level, and occupation. The clinical and HIV-related variables captured respondents’ health status and engagement with HIV care services, which included years since HIV diagnosis, HIV clinical stage, TB symptom screening, and duration on ART. Knowledge regarding TPT was assessed through structured questions covering its purpose, eligibility, benefits, and duration. Responses were scored and categorized into poor knowledge and good knowledge based on a predefined threshold. This variable measured whether respondents’ awareness and understanding of TPT influenced their likelihood of initiation.

### Definition of variables

Several clinical variables were defined in line with the national HIV and TB guidelines of the GHS and the corresponding WHO recommendations [13]. TB screening was performed using the WHO four-symptom screen (current cough, fever, night sweats, and unintentional weight loss). Clients who screened positive on any of these symptoms were regarded as having presumptive TB and were referred for further evaluation, including bacteriological testing with GeneXpert MTB/RIF and/or chest radiography, in keeping with the national TB diagnostic algorithm. Active TB was therefore excluded on the basis of a negative four-symptom screen and, where indicated, negative confirmatory investigations. Respondents found to have active TB were commenced on TB treatment rather than preventive therapy and were not eligible for TPT. Eligibility for TPT was accordingly defined as being a person living with HIV in care who had a negative TB symptom screen (no current evidence of active TB), was not already on TB treatment, had not previously completed a course of TPT, and had no documented contraindication to preventive therapy.

HIV clinical stage was based on the WHO clinical staging system (stages 1 to 4) as documented in the patient’s clinical folder by the attending clinician. CD4 cell counts and viral load results were not consistently documented in the records reviewed and were therefore not analysed in this study. With respect to the preventive regimen, isoniazid preventive therapy (a six-month course of isoniazid) was the predominant TPT regimen available in the participating facilities during the study period, while the shorter rifapentine-based regimens (such as 3HP) were not yet routinely available. The regimen was therefore essentially uniform across respondents and was not included as a variable in the regression model.

### Data analysis

Data collected were exported into Stata version 17.0 for processing and analysis. Prior to analysis, the dataset underwent rigorous cleaning procedures. This included checking for completeness, verifying out-of-range values, and assessing internal consistency across related variables. Variables were appropriately coded, and categorical variables were recoded where necessary to ensure suitability for analysis. Continuous variables were examined for normality using summary statistics and graphical methods.

Descriptive statistical analyses were first performed to summarize respondents’ characteristics. Categorical variables were presented as frequencies and percentages, while continuous variables were summarized using means and standard deviations. These descriptive results provided an overview of socio-demographic characteristics, clinical information, and healthcare system-related information. To identify factors associated with TPT initiation among PLHIV, inferential analysis was performed using logistic regression. Initially, bivariate (unadjusted) logistic regression analyses were performed to assess the independent association between each explanatory variable and the outcome variable (TPT initiation). Crude odds ratios (cORs), 95% confidence intervals (CIs), and corresponding p-values were generated. Variables with a test p-value of less than 0.05 at the bivariate level were considered eligible for inclusion in the multivariable logistic regression model. A multivariable logistic regression model was then fitted to determine the independent predictors of TPT initiation while controlling for potential confounding factors. Adjusted odds ratios (aORs), 95% confidence intervals, and p-values were reported. Statistical significance at the multivariable level was set at *p* < 0.05.

### Quality control

To ensure the data collected was of high quality and the instruments were error-free, pretesting was conducted at VRH among 15 eligible respondents who shared the same characteristics as those who participated in the main study. This was done to ensure the validity and reliability of the data collection instrument. Also, research assistants were trained to ensure they had an in-depth understanding of the data collection instrument as well as familiarize themselves with it.

## Results

### Sociodemographic characteristics of PLHIV

Table 1 presents the sociodemographic characteristics of the 806 respondents included in the study. The mean age of the respondents was 44.7 years (SD ± 13.0). The results indicate that the majority of respondents, 224 (27.8%), were between 41 and 50 years old, and the majority, 599 (74.3%), were females. Ewe was the predominant ethnicity among the respondents, 70.8% (571). With regards to religion, the majority, 661 (82%), were Christians, and most, 373 (46.3%), were married. About one-fourth, 216 (26.8%) of the study respondents had formal education up to Junior High School. Trading was the most common occupation among the respondents, 377 (46.8%). (Table 1)

**Table 1:** Sociodemographic characteristics of PLHIV

Variables	Frequency (*N* = 806)	Percentage (%)
Mean Age, SD	(44.7 ± 13.0)	
Age (years)		
18–30	117	14.4
31–40	193	24.0
41–50	224	27.8
51–60	170	21.1
> 61	102	12.6
Gender		
Female	599	74.3
Male	207	25.7
Ethnicity		
Akan	117	14.6
Ewe	571	70.8
Ga-dangbe	9	1.1
Guan	54	6.7
Mole Dagbani	8	1.0
Others^a^	47	5.8
Religion		
Christian	661	82.0
Muslim	52	6.5
Traditionalist	93	11.5
Marital status		
Single	161	20.0
Co-habiting	20	2.5
Married	373	46.3
Divorced/Separated	122	15.1
Widow	130	16.1
Level of education		
No formal education	169	21.0
Basic/Primary education	195	24.2
JHS^b^ education	216	26.8
Secondary education	182	22.5
Tertiary	44	5.5
Occupation		
Apprentice	49	6.1
Farmer	152	18.9
Government worker	46	5.7
Student	39	4.8
Trader	377	46.8
Others^c^	118	14.6
None	25	3.1
Health facility		
KSMH^d^	403	50.0
VRH^e^	403	50.0

^a^Others includes Artisans, skilled laborers, businessmen, and security personnel, ^b^Junior High School, ^c^Others include shoemakers, carpenters, etc., ^d^Ketu South Municipal Hospital, ^e^Volta Regional Hospital

### Clinical records of PLHIV

Table 2 presents the health records of the respondents. Most respondents, 608 (75.4%), had been diagnosed with HIV for one year or more, while 198 (24.6%) had been diagnosed for less than one year. Regarding HIV classification, the largest group was Stage 1, with 352 respondents (43.7%), followed by Stage 2, 267 (33.1%). Stage 3 accounted for 187 respondents (23.2%).

In relation to years on ART, the majority of the respondents, 630 (78.2%), had been on ART for one year or more. Among the respondents, 700 (86.8%) had documented as being screened for TB. Among the 700 who were screened, 620 (88.6%) were eligible to be initiated on TPT. **(**Table 2**)**

**Table 2:** Clinical records of PLHIV

Variable	Frequency (*N* = 806)	Percentage (%)
Years of HIV Diagnosis		
Less than a year	198	24.6
One year or more	608	75.4
HIV Classification		
Stage 1	352	43.7
Stage 2	267	33.1
Stage 3	187	23.2
Years on ART		
Less than a year	176	21.8
One year or more	630	78.2
Screened for TB		
No	106	13.2
Yes	700	86.8
Eligible for TPT (*n* = 700)		
No	80	11.4
Yes	620	88.6
Initiated TPT (*n* = 620)		
No	384	61.9
Yes	236	38.1

### Proportion of PLHIV initiated on TPT

**Fig. 2:**
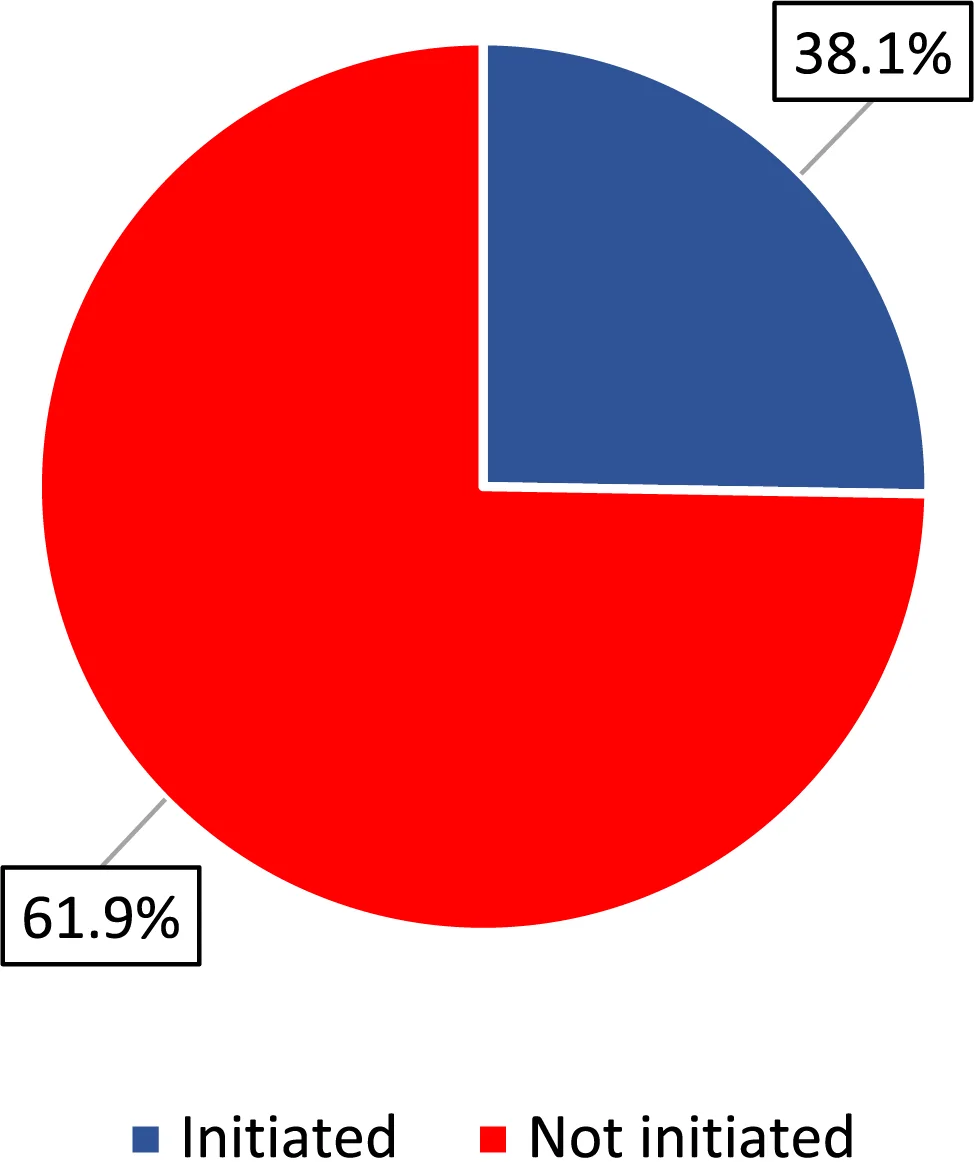
Proportion of PLHIV initiated on TPT

Figure 2 presents the proportion of PLHIV initiated on TPT. The results indicate that among respondents eligible for TPT, the majority, 384/620 (61.9%), of the respondents were not initiated on TPT. **(**Fig. 2**)**

### Knowledge of PLHIV on TPT

**Fig. 3:**
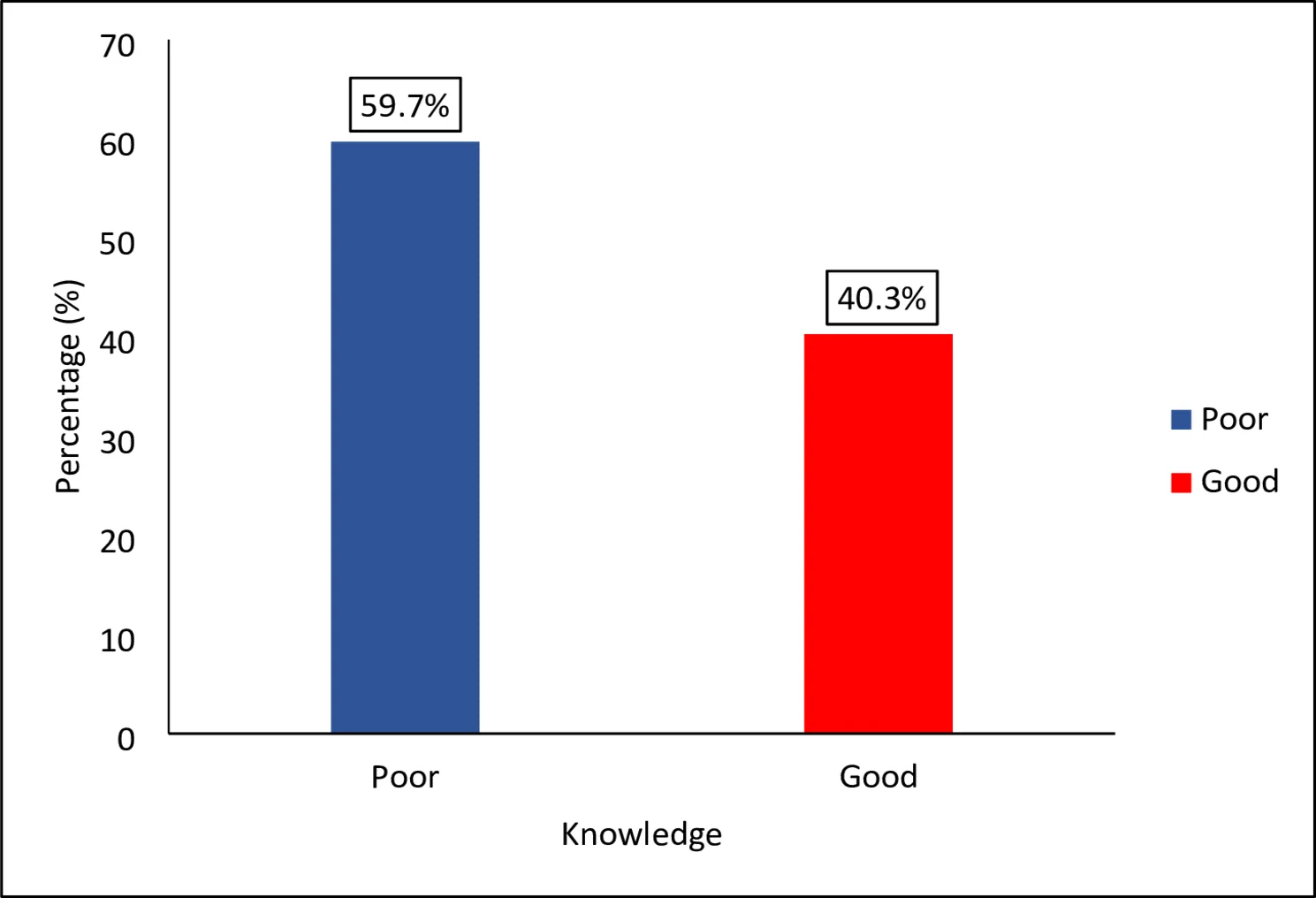
Knowledge of PLHIV on TPT

Figure 3 below illustrates the respondents’ knowledge level regarding TPT. The results show that less than half of the total respondents (40.3%) had good knowledge of TPT, while the majority (59.7%) had poor knowledge of TPT. (Fig. 3)

### Healthcare system-related factors on TPT initiation

The result shows that the majority, 607 (75.3%) of the respondents, said they did not have to make a payment for TPT services. When asked if they faced challenges in accessing TPT, only a few of the respondents, 65 (8.1%), said they faced challenges, while the majority, 452 (56.1%), said they did not know if there were any challenges or not. Among those who faced challenges, the majority, 44 (67.7%), cited distance to the health facility as the main issue, followed by the cost of transportation, 39 (60.0%). A few of the respondents, 76 (9.4%), said they had experienced adverse drug reactions in relation to TPT. Only a few of the respondents, 229 (28.4%), said they faced some challenges in the healthcare system in terms of accessing TPT services, with the majority citing limited availability of drugs, 204 (89.1%), and poor health education, 103 (45.0%), as the main health system barriers. Almost half of the respondents, 349 (43.3%), said they did not receive adequate information from the health workers regarding TPT services. (Table 3)

**Table 3:** Healthcare system-related factors on TPT initiation

Variables	Frequency (*N* = 806)	Percentage (%)
Make payment for TPT services		
Yes	0	0.0
No	607	75.3
Don’t know	199	24.7
Face challenges in accessing TPT		
Yes	65	8.1
No	289	35.8
Don’t know	452	56.1
Challenges faced in accessing TPT (Multiple choice *n* = 65)		
Poor health literacy	9	13.9
Lack of information sources	17	26.2
Distance to the health facility	44	67.7
Cost of the hospital services	17	26.2
Cost of transportation	39	60.0
Experienced adverse drug reactions in relation to TPT		
Yes	76	9.4
No	145	18.0
Don’t know	585	72.6
Face challenges in the healthcare system when accessing TPT services		
Yes	229	28.4
No	136	16.9
Don’t know	441	54.7
Health system barriers faced in accessing TPT (*n* = 229)		
Poor health education	103	45.0
Limited availability of drugs	204	89.1
Poor quality of TPT services	86	37.6
Health service delay	43	18.8
Receives adequate information from the health workers		
Yes	457	56.7
No	349	43.3

### Predictors of TPT initiation among PLHIV

Table 4 presents the factors associated with TPT initiation among the 620 eligible respondents. At the bivariate level, marital status (*p* = 0.027), knowledge (*p* < 0.001), years since HIV diagnosis (*p* < 0.001), HIV classification (*p* < 0.001), and years on ART (*p* < 0.001) were each significantly associated with TPT initiation. After adjusting for the other variables in the model, only knowledge (*p* < 0.001), years since HIV diagnosis (*p* < 0.001), HIV classification (*p* < 0.001), and marital status (*p* = 0.038) remained statistically significantly associated with TPT initiation. Respondents who had good knowledge of TPT were about 7 times more likely to be initiated compared with those who had poor knowledge (aOR = 6.79, 95% CI 4.69–9.83, *p* < 0.001). Those who had been diagnosed with HIV for one year or more were 4 times as likely to be initiated as those diagnosed for less than a year (aOR = 4.18, 95% CI 1.74–10.04, *p* = 0.001). Respondents in HIV clinical stage 2 were 66% less likely to be initiated than those in stage 1 (aOR = 0.34, 95% CI 0.20–0.58, *p* < 0.001). With respect to marital status, married respondents were 40% less likely to be initiated on TPT compared with single respondents (aOR = 0.60, 95% CI 0.37–0.97, *p* = 0.038) (Table 4).

**Table 4:** Predictors of TPT initiation among PLHIV eligible for TPT (*N* = 620)

Variables	TPT initiation (*N* = 620)		cOR (95% CI) *p*-value	aOR (95% CI) *p*-value
	No (%)	Yes (%)		
Age				
18–30	50(53.8%)	43(46.2%)	**Ref.**	
31–40	88(59.5%)	60(40.5%)	0.79(0.47–1.34) 0.385	
41–50	110(64.7%)	60(35.3%)	0.63(0.38–1.06) 0.083	
51–60	82(63.1%)	48(36.9%)	0.68(0.40–1.17) 0.164	
> 61	54(68.4%)	25(31.6%)	0.54(0.29–1.01) 0.052	
Gender				
Female	285(62.1%)	174(37.9%)	**Ref.**	
Male	99(61.5%)	62(38.5%)	1.43(0.72–1.46) 0.942	
Ethnicity				
Akan	54(60.0%)	36(40.0%)	**Ref.**	
Ewe	276(63.2%)	161(36.8%)	0.88(0.55–1.39) 0.573	
Ga-dangbe	5(71.4%)	2(28.6%)	0.60(0.11–3.27) 0.555	
Guan	26(61.9%)	16(38.1%)	0.92(0.44–1.96) 0.835	
Mole-Dagbani	5(83.3%)	1(16.7%)	0.30(0.03–2.67) 0.281	
Others	18(47.4%)	20(52.6%)	1.67(0.78–3.57) 0.190	
Religion				
Christian	325(62.3%)	197(37.7%)	**Ref.**	
Muslim	24(54.5%)	20(45.5%)	1.38(0.74–2.56) 0.313	
Traditionalist	35(64.8%)	19(35.2%)	0.90(0.50–1.61) 0.713	
Marital status				
Single	75(53.6%)	65(46.4%)	**Ref.**	**Ref.**
Co-habiting	12(75.0%)	4(25.0%)	0.39(0.12–1.25) 0.113	0.41(0.12–1.39) 0.152
Married	175(64.8%)	95(35.2%)	0.63(0.41–0.95) 0.027*	0.60(0.37–0.97) 0.038*
Divorced/Separated	60(59.4%)	41(40.6%)	0.79(0.47–1.32) 0.367	0.82(0.46–1.46) 0.498
Widowed	62(66.7%)	31(33.3%)	0.58(0.33–0.99) 0.048*	0.54(0.29–0.99) 0.056
Level of education				
No formal education	80(61.5%)	50(38.5%)	**Ref.**	
Basic/Primary education	92(63.0%)	54(37.0%)	0.94(0.58–1.53) 0.800	
JHS education	100(62.5%)	60(37.5%)	0.96(0.60–1.55) 0.867	
SHS/SSS/O level/A level education	85(59.0%)	59(41.0%)	1.11(0.68–1.80) 0.670	
Tertiary education	27(67.5%)	13(32.5%)	0.77(0.36–1.63) 0.495	
Occupation				
None	15(71.4%)	6(28.6%)	**Ref.**	
Apprentice	20(48.8%)	21(51.2%)	2.63(0.85–8.10) 0.093	
Farmer	70(58.8%)	49(41.2%)	1.75(0.64–4.82) 0.280	
Government worker	25(71.4%)	10(28.6%)	1.00(0.30–3.31) 1.000	
Student	15(46.9%)	17(53.1%)	2.83(0.88–9.16) 0.082	
Trader	180(63.8%)	102(36.2%)	1.42(0.53–3.76) 0.485	
Others	59(65.6%)	31(34.4%)	1.31(0.46–3.72) 0.608	
Knowledge				
Poor	290(80.8%)	69(19.2%)	**Ref.**	**Ref.**
Good	94(36.0%)	167(64.0%)	7.47(5.19–10.75) < 0.001*	6.79(4.69–9.83) < 0.001*
Years of HIV Diagnosis				
Less than a year	60(88.2%)	8(11.8%)	**Ref.**	**Ref.**
One year or more	324(58.7%)	228(41.3%)	5.28(2.48–11.25) < 0.001*	4.18(1.74–10.04) 0.001*
HIV Classification				
Stage 1	210(54.8%)	173(45.2%)	**Ref.**	**Ref.**
Stage 2	105(80.8%)	25(19.2%)	0.29(0.18–0.47) < 0.001*	0.34(0.20–0.58) < 0.001*
Stage 3	69(64.5%)	38(35.5%)	0.67(0.43–1.04) 0.073	0.59(0.36–0.98) 0.052*
Years on ART				
Less than a year	70(87.5%)	10(12.5%)	**Ref.**	**Ref.**
One year or more	314(58.1%)	226(41.9%)	5.04(2.54–9.99) < 0.001*	1.39(0.60–3.23) 0.443

*Variables that attained statistical significance at *P* < 0.05

## Discussion

### Low Uptake of TPT among PLHIV

This study found that only 38.1% of PLHIV were initiated on TPT, indicating substantial gaps in the delivery of preventive TB services within HIV care. This level of uptake is low considering that the WHO strongly recommends systematic provision of TPT to all eligible PLHIV to reduce the risk of developing active tuberculosis [18]. Despite global progress in scaling up TPT, the 2023 WHO Global Tuberculosis Report highlights that millions of eligible PLHIV still do not receive preventive therapy [18].

Our findings are consistent with studies from several sub-Saharan African settings, where TPT initiation remains suboptimal despite national adoption of WHO guidelines [19, 20]. In contrast, countries that have achieved higher TPT coverage have implemented strong integration of TB and HIV services, reliable drug supply systems, and active patient follow-up mechanisms. The low uptake observed in this study, therefore, suggests implementation gaps rather than policy absence, highlighting the need for strengthened service delivery within HIV clinics.

### Structural and health system barriers

Although most respondents (75.3%) reported that they did not pay for TPT services, consistent with WHO recommendations that preventive therapy should be provided free of charge, indirect access barriers remained. Distance to health facilities and transportation costs were the main challenges reported by respondents. Similar findings have been reported in other low- and middle-income countries, where indirect costs such as travel expenses and time away from work continue to influence access to HIV and TB services even when treatment is free [20].

Health system barriers were also evident. Just over a quarter of respondents reported challenges related to drug availability and inadequate health education. Drug stock-outs have been widely identified as a key barrier to scaling up TPT in high-burden settings [19]. Inconsistent availability of preventive therapy medications may undermine both patient confidence and provider willingness to initiate treatment. Furthermore, nearly two-fifths of respondents reported not receiving adequate information about TPT from healthcare providers. This finding is important because inadequate communication may limit patient understanding of preventive therapy and reduce willingness to initiate treatment. Strengthening drug supply systems and provider-led patient education, therefore, remains essential for improving TPT coverage.

### Individual and social determinants of TPT initiation

This study identified several individual and social factors associated with TPT initiation. Knowledge emerged as the strongest predictor, with individuals who had good knowledge about TPT being significantly more likely to initiate the therapy. This finding aligns with previous studies demonstrating that patient awareness and understanding play a critical role in the uptake of preventive interventions [21]. The WHO also identifies patient education and counseling as key components of TPT scale-up strategies [22]. Without adequate knowledge of the benefits, duration, and safety of preventive therapy, patients may be reluctant to initiate treatment. Strengthening structured health education and counseling within HIV clinics may therefore be an effective strategy to improve TPT initiation.

Marital status was also associated with TPT initiation, with married individuals being less likely to initiate the therapy compared to single respondents. Although marital support is often associated with improved HIV treatment outcomes, relational dynamics may also introduce challenges related to stigma, disclosure, or decision-making autonomy in some contexts. Previous research has shown that concerns about HIV-related stigma within intimate relationships can negatively influence engagement with HIV services [20]. These findings suggest that social and relational factors may influence acceptance of preventive therapies and highlight the potential value of couple-based counseling and stigma reduction strategies within HIV programs. Nonetheless, the association between marital status and preventive therapy uptake has been inconsistent across settings, and the present cross-sectional analysis cannot establish the mechanism underlying this finding. We therefore recommend that a dedicated follow-up study, ideally incorporating qualitative methods, be conducted to explore how relationship dynamics, HIV status disclosure, and household decision-making influence TPT initiation among married PLHIV, so that appropriately tailored counseling strategies can be developed.

### Influence of HIV care engagement and clinical status

Duration since HIV diagnosis was another important predictor of TPT initiation. Individuals who had been diagnosed with HIV for one year or more demonstrated substantially higher odds of initiating TPT than those recently diagnosed. This suggests that longer engagement in HIV care enhances trust in healthcare providers, familiarity with treatment protocols, and exposure to repeated counseling messages. Newly diagnosed individuals often prioritize initiation of antiretroviral therapy and psychological adjustment to their diagnosis, which may delay acceptance of additional preventive treatments. Integrating TPT counseling earlier in the HIV care pathway may therefore help reduce delays in initiation.

Clinical stage of HIV infection also influenced TPT initiation, with lower uptake observed among individuals in Stage 2 and Stage 3 compared to those in Stage 1. Although WHO guidelines recommend TPT for eligible PLHIV regardless of clinical stage once active TB has been excluded, healthcare providers may exercise caution when managing patients with more advanced disease due to coexisting opportunistic infections or competing clinical priorities. Nevertheless, this pattern raises concerns about equitable access to preventive therapy across clinical stages, underscoring the importance of standardized eligibility assessment and consistent guideline implementation. It is important to note that both facilities operate under the same national HIV and TB guidelines [13], which recommend TPT for all eligible PLHIV once active TB has been excluded, irrespective of WHO clinical stage. The lower initiation observed at more advanced stages, therefore, reflects differences in implementation and provider caution rather than the use of a different guideline. In this context, advanced HIV disease (AHD), generally defined in adults as WHO clinical stage 3 or 4, or a CD4 count below 200 cells/mm³, represents the group in which competing clinical priorities are most likely to delay preventive therapy. Because CD4 counts and viral load results were not consistently documented in the records reviewed, we were unable to report the proportion of respondents who were virally suppressed; HIV staging in this study was therefore based on the WHO clinical staging system, and the inability to triangulate clinical stage with immunological and virological markers should be borne in mind when interpreting these findings.

### Implications for TPT scale-up

Taken together, these findings suggest that low TPT initiation among PLHIV is driven by a combination of knowledge-related, individual, social, and health system factors. While patient awareness appears to be a key driver of initiation, structural barriers such as transportation costs, health system constraints including drug availability, and social influences related to marital dynamics also play important roles. Strengthening patient education, improving healthcare provider communication, ensuring consistent drug supply, and addressing social barriers may substantially improve TPT uptake within HIV care programs.

## Conclusion

This study identified a substantial gap in the initiation of TPT among PLHIV, with fewer than one-third of eligible individuals initiating therapy despite global recommendations for universal provision. Knowledge emerged as the strongest predictor of TPT initiation, while marital status, duration since HIV diagnosis, and HIV clinical stage also influenced uptake. Although direct financial barriers were minimal, structural and health system challenges such as distance to health facilities, inconsistent drug availability, and inadequate patient education continue to limit effective implementation. These findings underscore the need for strengthened patient education, improved provider–patient communication, and more reliable drug supply systems within HIV care services.

Addressing these implementation gaps is essential for expanding TPT coverage, reducing tuberculosis risk among PLHIV, and accelerating progress toward United Nations Sustainable Development Goal (SDG) 3, particularly SDG 3.3, which aims to end the epidemics of AIDS and tuberculosis by 2030.

## Limitations

This study combined self-reported information (socio-demographic characteristics, knowledge of TPT, perceived barriers, and experiences with TPT services) with clinical data abstracted from patients’ folders and ART registers (TPT initiation, WHO clinical stage, duration since HIV diagnosis, duration on ART, and TB screening status). The self-reported components are susceptible to social desirability and recall bias; however, the outcome and the key clinical variables were obtained from documented records, which limits this bias. In addition, CD4 cell counts and viral load results were not consistently available in the records reviewed, so we were unable to report immunological or virological status (for example, the proportion of respondents who were virally suppressed) or to use these markers to refine HIV staging. Finally, there was limited generalizability due to a relatively homogeneous sample of PLHIV drawn from only two ART clinics within the Volta region, which may not represent the broader PLHIV population.

## Supplementary Information

Supplementary Material 1.

## Data Availability

All relevant data relating to the manuscript are available upon request. Requests regarding the data used for this study could be made through the corresponding author.
