# Supplementary material for: Predictors of tuberculosis preventive therapy initiation among people living with HIV in the Volta region, Ghana
Source: BMC Public Health. 2026 Jul 23;26:3000. doi: 10.1186/s12889-026-28511-w (PMC13679220; doi:10.1186/s12889-026-28511-w)
Supplement: Supplementary file 1 [file 12889_2026_28511_MOESM1_ESM.docx]

**RESEARCH QUESTIONNAIRE**

**Predictors of Tuberculosis Preventive Therapy (TPT) Initiation among People Living with HIV**

**in the Volta Region, Ghana**

***Interviewer-administered structured questionnaire***

**INSTRUCTIONS TO INTERVIEWERS:**

Please read each question clearly to the respondent and record their response accurately. For multiple-choice questions, read all options before recording the response. All clinical data in Section 2 should be verified against the patient's medical/clinical records.

Participant ID: ____________	Date of Interview: __ / __ / ______	Interviewer Code: _______

| **S/N** | **Question** | **Response** |
| --- | --- | --- |
| **SECTION 1: Sociodemographic Characteristics** | | |
| **Q1** | What is your current age (in years)? | ............... |
| **Q2** | What is your gender? | 1. Female  2. Male |
| **Q3** | What is your ethnic group/affiliation? | 1. Akan  2. Ewe  3. Ga-Dangbe  4. Guan  5. Mole-Dagbani  6. Other (specify): ___ |
| **Q4** | What is your religion? | 1. Christianity  2. Islam  3. Traditional religion |
| **Q5** | What is your current marital status? | 1. Single (never married)  2. Co-habiting  3. Married  4. Divorced / Separated  5. Widowed |
| **Q6** | What is the highest level of education you have completed? | 1. No formal education  2. Basic / Primary education  3. JHS education  4. Secondary education  5. Tertiary education |
| **Q7** | What is your current occupation or main source of livelihood? | 1. Apprentice  2. Farmer  3. Government worker  4. Student  5. Trader  6. None  7. Other (specify): ___ |
| **Q8** | Which health facility are you currently enrolled in for HIV care? | 1. Keta South Municipal Hospital (KSMH)  2. Volta Regional Hospital (VRH) |
| **SECTION 2: Clinical Records of People Living with HIV (PLHIV)** | | |
| **Q9** | How long ago were you diagnosed with HIV? | 1. Less than 1 year  2. One year or more |
| **Q10** | What is your current HIV clinical stage as documented in your medical record? | 1. Stage 1  2. Stage 2  3. Stage 3 |
| **Q11** | How long have you been on antiretroviral therapy (ART)? | 1. Less than 1 year  2. About 1 year  3. More than 1 year |
| **Q12** | Have you ever been screened for tuberculosis (TB) at this health facility? | 1. Yes  2. No |
| **Q13** | After your TB screening, were you found to be eligible for Tuberculosis Preventive Therapy (TPT)? | 1. Yes  2. No |
| **Q14** | Did you initiate (start) Tuberculosis Preventive Therapy (TPT)? | 1. Yes  2. No |
| ***Questions Q9–Q14 are to be verified against the patient's medical/clinical records.*** | | |
| **SECTION 3: Knowledge of PLHIV on Tuberculosis Preventive Therapy (TPT)** | | |
| **Q15** | Have you ever heard of Tuberculosis Preventive Therapy (TPT)? | 1. Yes  2. No |
| **Q16** | In your own understanding, what is the purpose of Tuberculosis Preventive Therapy (TPT)? | 1. To prevent the development of active TB disease in people at risk  2. To treat active tuberculosis disease  3. To treat HIV infection  4. I do not know |
| **Q17** | Who do you think is eligible to receive Tuberculosis Preventive Therapy (TPT)? | 1. People living with HIV (PLHIV)  2. People who have had close contact with someone with TB  3. Both PLHIV and TB contacts  4. I do not know |
| **Q18** | Which of the following medicines do you know is/are used for Tuberculosis Preventive Therapy (TPT)? | 1. Isoniazid (INH) only  2. Rifapentine and Isoniazid (3HP)  3. Both isoniazid and rifapentine-based regimens  4. I do not know |
| **Q19** | How long should a course of Tuberculosis Preventive Therapy (TPT) last? | 1. Less than 1 month  2. 1–3 months  3. 6–12 months  4. I do not know |
| **Q20** | How is Tuberculosis Preventive Therapy (TPT) administered? | 1. Oral tablets taken daily  2. Weekly oral tablets  3. Injection  4. I do not know |
| **Q21** | Can a person living with HIV take Tuberculosis Preventive Therapy (TPT) at the same time as antiretroviral therapy (ART)? | 1. Yes, they can be taken together  2. No, they cannot be taken together  3. I do not know |
| **Q22** | Does Tuberculosis Preventive Therapy (TPT) treat active tuberculosis disease? | 1. Yes  2. No |
| **Q23** | Do you know that people living with HIV (PLHIV) are at a higher risk of developing tuberculosis (TB) compared to HIV-negative individuals? | 1. Yes  2. No |
| **Q24** | Do you believe that taking Tuberculosis Preventive Therapy (TPT) can reduce the chance of a person living with HIV developing active TB? | 1. Yes  2. No |
| **Q25** | Are you aware of any common side effects of Tuberculosis Preventive Therapy (TPT)? | 1. Yes  2. No |
| **Q26** | Where can Tuberculosis Preventive Therapy (TPT) medicines be obtained? | 1. At a public health facility/ART clinic  2. At a private pharmacy  3. At any hospital  4. I do not know |
| *Note: Section 3 measures specific knowledge about TPT. Questions were adapted and contextualized from:  1. Palacios CF, Moll AP, Shrestha R, Kompala T, Davis JL, Charalambous S, et al. Evaluating community knowledge of tuberculosis preventive therapy in rural South Africa. BMC Public Health. 2025 Mar 7;25(1):918. Available from:* [*https://doi.org/10.1186/s12889-025-21719-2*](https://doi.org/10.1186/s12889-025-21719-2)  *2. Mindachew M, Deribew A, Tessema F, Biadgilign S. Predictors of adherence to isoniazid preventive therapy among HIV positive adults in Addis Ababa, Ethiopia. BMC Public Health [Internet]. 2011 Dec 1;11(1):916. Available from:* [*https://doi.org/10.1186/1471-2458-11-916*](https://doi.org/10.1186/1471-2458-11-916)  *3. World Health Organization. WHO consolidated guidelines on tuberculosis: Module 1: Prevention-infection prevention and control. World Health Organization; 2022 Aug 12.* | | |
| **SECTION 4: Healthcare System-Related Factors on TPT Initiation** | | |
| **Q27** | Do you make any payment for Tuberculosis Preventive Therapy (TPT) services at this facility? | 1. Yes  2. No  3. I do not know |
| **Q28** | Have you ever faced any challenges in accessing Tuberculosis Preventive Therapy (TPT) services? | 1. Yes  2. No  3. I do not know |
| **Q29** | If yes (Q28), what challenges did you face in accessing TPT? **(Select all that apply)** | 1. Poor health literacy/difficulty understanding information about TPT  2. Lack of information sources about TPT  3. Long distance to the health facility  4. High cost of hospital services  5. High cost of transportation to the health facility  6. Other (specify): ___ |
| **Q30** | Have you ever experienced any adverse drug reactions (side effects) in relation to Tuberculosis Preventive Therapy (TPT)? | 1. Yes  2. No  3. I do not know |
| **Q31** | Have you encountered challenges within the healthcare system when trying to access TPT services? | 1. Yes  2. No  3. I do not know |
| **Q32** | If yes (Q31), what healthcare system barriers did you experience? (Select all that apply) | 1. Poor health education / inadequate counselling from healthcare workers  2. Limited availability (stock-outs) of TPT drugs at the facility  3. Poor quality of TPT services  4. Long health service delays (long waiting times)  5. Other (specify): ___ |
| **Q33** | Do you receive adequate information about Tuberculosis Preventive Therapy (TPT) from the healthcare workers at this facility? | 1. Yes  2. No  3. I do not know |
| *Note: Section 4 items were adapted from:*  *1. Roscoe C, Lockhart C, De Klerk M, Baughman A, Agolory S, Gawanab M, et al. Evaluation of the uptake of tuberculosis preventative therapy for people living with HIV in Namibia: a multiple methods analysis. BMC Public Health [Internet]. 2020 Dec 1;20(1):1838. Available from:* [*https://doi.org/10.1186/s12889-020-09902-z*](https://doi.org/10.1186/s12889-020-09902-z)  *2. Getahun H, Granich R, Sculier D, Gunneberg C, Blanc L, Nunn P, et al. Implementation of isoniazid preventive therapy for people living with HIV worldwide: barriers and solutions. AIDS. 2010 Nov 1;24(Suppl 5):S57–65. Available from:* [*https://doi.org/10.1097/01.aids.0000391023.03037.1f*](https://doi.org/10.1097/01.aids.0000391023.03037.1f) | | |

**List of references**

1. Palacios CF, Moll AP, Shrestha R, Kompala T, Davis JL, Charalambous S, et al. Evaluating community knowledge of tuberculosis preventive therapy in rural South Africa. BMC Public Health. 2025 Mar 7;25(1):918. Available from: <https://doi.org/10.1186/s12889-025-21719-2>

2. Mindachew M, Deribew A, Tessema F, Biadgilign S. Predictors of adherence to isoniazid preventive therapy among HIV positive adults in Addis Ababa, Ethiopia. BMC Public Health [Internet]. 2011 Dec 1;11(1):916. Available from: <https://doi.org/10.1186/1471-2458-11-916>

3. World Health Organization. WHO consolidated guidelines on tuberculosis: Module 1: Prevention-infection prevention and control. World Health Organization; 2022 Aug 12.

4. Getahun H, Granich R, Sculier D, Gunneberg C, Blanc L, Nunn P, et al. Implementation of isoniazid preventive therapy for people living with HIV worldwide: barriers and solutions. AIDS. 2010 Nov 1;24(Suppl 5):S57–65. Available from: <https://doi.org/10.1097/01.aids.0000391023.03037.1f>

5. Roscoe C, Lockhart C, De Klerk M, Baughman A, Agolory S, Gawanab M, et al. Evaluation of the uptake of tuberculosis preventative therapy for people living with HIV in Namibia: a multiple methods analysis. BMC Public Health. 2020 Dec 1;20(1):1838. Available from: <https://doi.org/10.1186/s12889-020-09902-z>
